# Source/Drain Trimming Process to Improve Gate-All-Around Nanosheet Transistors Switching Performance and Enable More Stacks of Nanosheets

**DOI:** 10.3390/mi13071080

**Published:** 2022-07-08

**Authors:** Kun Chen, Jingwen Yang, Tao Liu, Dawei Wang, Min Xu, Chunlei Wu, Chen Wang, Saisheng Xu, David Wei Zhang, Wenchao Liu

**Affiliations:** 1 State Key Laboratory of ASIC and System, School of Microelectronics, Fudan University, Shanghai 200433, China; chenk18@fudan.edu.cn (K.C.); 18112020011@fudan.edu.cn (J.Y.); tliu14@fudan.edu.cn (T.L.); 20112020120@fudan.edu.cn (D.W.); chen_w@fudan.edu.cn (C.W.); ssxu@fudan.edu.cn (S.X.); 2 Shanghai Integrated Circuit Manufacturing Innovation Center Co., Ltd., Shanghai 200433, China; 3 Zhangjiang Fudan International Innovation Center, Shanghai 200433, China; 4 Primarius Technologies Co., Ltd., Shanghai 201306, China; liuwc@khai-long.com

**Keywords:** gate-all-around (GAA), nanosheet (NS), S/D stressor, channel stress enhancement

## Abstract

A new S/D trimming process was proposed to significantly reduce the parasitic RC of gate-all-around (GAA) nanosheet transistors (NS-FETs) while retaining the channel stress from epitaxy S/D stressors at most. With optimized S/D trimming, the 7-stage ring oscillator (RO) gained up to 27.8% improvement of delay with the same power consumption, for a 3-layer stacked GAA NS-FETs. Furthermore, the proposed S/D trimming technology could enable more than 4-layer vertical stacking of nanosheets for GAA technology extension beyond 3 nm CMOS technology.

## 1. Introduction

Gate-All-Around (GAA) Nanosheet (NS) transistor is the most promising candidate for 3 nm node and beyond, owing to its superior electrostatics compared to FinFET [1]. For GAA NS-FETs technology, vertical stacking architecture with multiple parallel channels is the key to boost drive current capability at a given footprint [2]. However, GAA technology faces some critical fabrication challenges such as channel release, formation of inner-spacer, and epitaxy growth of source/drain (S/D) SiGe stressor. The channel release and inner-spacer require ultra-high selective SiGe etching to retain stacked Si channels integrity [3], and advanced ALD low-k dielectric deposition and precision etch to control inner-spacer thickness and uniformity [4]. In addition, for GAA, the main transport surface orientation changes from (110) to (100), which has higher electron mobility but lower hole mobility. Therefore, the epitaxy growth of S/D SiGe stressor becomes extremely important and challenging to achieve N/P current matching. Furthermore, the S/D parasitic RC would be a bottleneck for drive current boost due to normally large epitaxy S/D volume required for channel stress engineering [5]. However, how to balance the channel stress and S/D RC optimization, and its impacts on GAA NS-FETs have not been systematically investigation.

In this work, we present a new integration scheme of a self-align S/D trimming process to solve the trade-off between channel stress engineering and S/D RC optimization. Based on device and system TCAD studies, the proposed S/D trimming scheme offers superior switching performance with almost no sacrificing of transistor DC performance. Furthermore, this new integration scheme may provide a potential path for continuing track-height scaling and enable more vertical stacking of nanosheets.

## 2. S/D Epi Growth and Impact on GAA NS-FETS

### 2.1. Simulation Methodology

To evaluate realistic S/D process impact on GAA NS-FETs, a three-layer vertical stacking Si GAA nanosheets structure is selected for TCAD simulation. Key parameters of the assumed structure, including gate length of 12 nm, NS width of 20 nm, gate pitch of 44 nm, nanosheet thickness of 5 nm, spacing between nanosheets of 12 nm, and contact poly pitch of 44 nm, were adopted referring to the IRDS roadmap for 3 nm node.

3D full flow process simulation, from SiGe/Si super-lattice growth to M0 contact formation, is carried out by the Sentaurus Process simulator. The compressive Si${}_{0.7}$Ge${}_{0.3}$ sacrificial layers with initial in-plane biaxial stress of −2 GPa were assumed. Lattice Kinetic Monte Carlo (LKMC) model is employed to accurately simulate the epitaxial Si${}_{0.6}$Ge${}_{0.4}$ (p-FETs) S/D stressor growth.

Both drift–diffusion transport model and quantum potential model are employed in Sentaurus SDevice for DC performance simulation after structure generation. Low field ballistic mobility, auto-orientation inversion, accumulation layer mobility, and high field saturation velocity were also included to account for the electrical characteristics of the nanoscale device. The multi-valley electron and hole mobility model was enabled to calculate strain effects. The physical parameters of the baseline GAA Si NS-FET simulation models were carefully calibrated using experimental data [1]. The electrical behavior of simulated NS-FETs is modeled with the Primarius BSIMplus module. Inverter and Ring Oscillator (RO) are constructed for AC performance evaluation.

### 2.2. Stress Requirements and S/D RC Concern for NS-FETs

**Figure 1:**
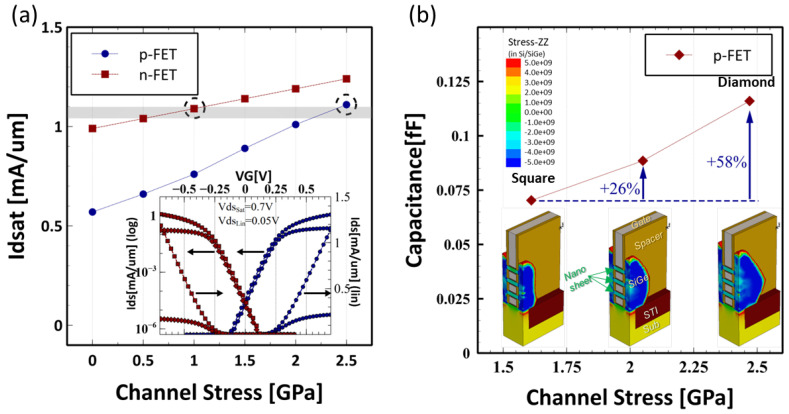
(**a**) Idsat response curve versus Channel stress for NS-FETs, with n-FET @ 1.0 GPa and p-FET @ 2.5 GPa, good N/P current matching is achieved; (**b**) capacitance versus stress for different S/D EPI sizes. The high stress EPI volume comes with capacitance penalty.

The majority of the surface area in FinFET is lateral (110)/<110>, while for NS-FET, the main transport surface becomes (100)/<110>. Because (100)/<110> orientation has higher electron mobility but lower hole mobility, it is much more difficult to achieve N/P current matching for 3 nm GAA NS-FETs technology. One of the most effective methods would be channel stress engineering by an epitaxial S/D stressor to boost carrier mobility. In order to investigate the stress engineering requirement for NS-FETs N/P current matching, transistor drive current Ion versus channel stress was simulated and plotted in Figure 1a for both n-FETs and p-FETs.

As clearly seen in Figure 1a, p-FET Idsat is only around half of n-FET Idsat without channel stress, but p-FET has much higher stress sensitivity than n-FET. As reported in Ref. [6], the channel would inherit 450–950 MPa tensile stress from the release of the sacrificial SiGe layer due to stress transfer. This favors n-FET and makes it very crucial to have higher channel stress in p-FET. As shown in the inset of Figure 1a, good N/P current matching could be realized with n-FET @ 1 GPa tensile stress and p-FET @ 2.5 GPa compressive stress, indicating it is extremely important to engineer the channel stress well, especially for p-FET.

As well known, S/D SiGe EPI volume strongly influences channel stress; therefore, LKMC EPI process simulation is performed to investigate the S/D SiGe EPI evolution and its effect on p-FET channel stress. As shown in Figure 1b, three selected points during S/D SiGe epitaxy growth are presented with a cut-away structure diagram to illustrate the S/D shape evolution, with corresponding stress and Cgg capacitance. Initially, the EPI growth was started on three small regions and evolve to a merged ‘Square’ shaped S/D, where the channel stress reaches around 1.6 GPa. As the epitaxy growth continues, the S/D shape finally becomes the characteristic ‘Diamond’ shape with the desired 2.5 GPa channel stress for p-FET to realize N/P current matching. However, this comes with a penalty of a 58% increase of Cgg, which raises a concern for the AC performance. As a result, the engineering between channel stress and parasitic RC would be critical to unlock the full potential of GAA NS-FETs technology.

## 3. Self-Align S/D Trim Scheme

### 3.1. S/D Trimming Process Flow

**Figure 2:**
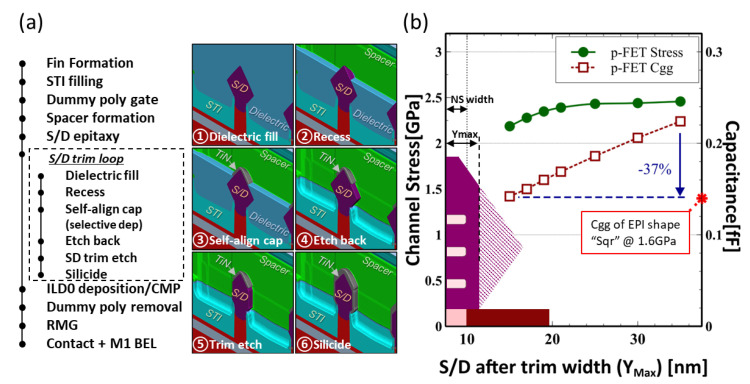
(**a**) Process details of self-aligned S/D trimming scheme and its relative position in the main flow with step by step process simulation animation; (**b**) average p-FET nanosheet channel stress and its corresponding capacitance as a function of its max width after trim.

To address the abovementioned trade-off issue between channel stress and parasitic RC, a new self-aligned S/D trimming integration scheme is proposed. This self-aligned trimming process after S/D EPI consists of (1) dielectric fill and pull back recess to expose the top of the S/D, (2) the exposed S/D parts undergo selective TiN deposition to form a self-aligned cap [7], (3) dielectric etch back, and (4) use the TiN as a hard-mask to trim off the side tips of ‘Diamond’ S/D structure [8]. The proposed integration flow is implemented and demonstrated in Sentaurus SProcess, as shown in Figure 2a, and the impact of trimming on NS-FETs channel stress and Cgg were evaluated as in Figure 2b. In the region of Ymax > 25 nm, the channel stress loss is almost negligible (<3%), even when Ymax∼15 nm, the channel stress loss is within 10%. At Ymax∼15 nm, which is approximately the same width of as-grown “Square” structure, the stress can retain to 2.18 GPa compared to 1.6 GPa for the as-grown one, while the parasitic capacitance can be reduced by 37%.

### 3.2. The Impact of S/D Trimming on Electrical Behavior

**Figure 3:**
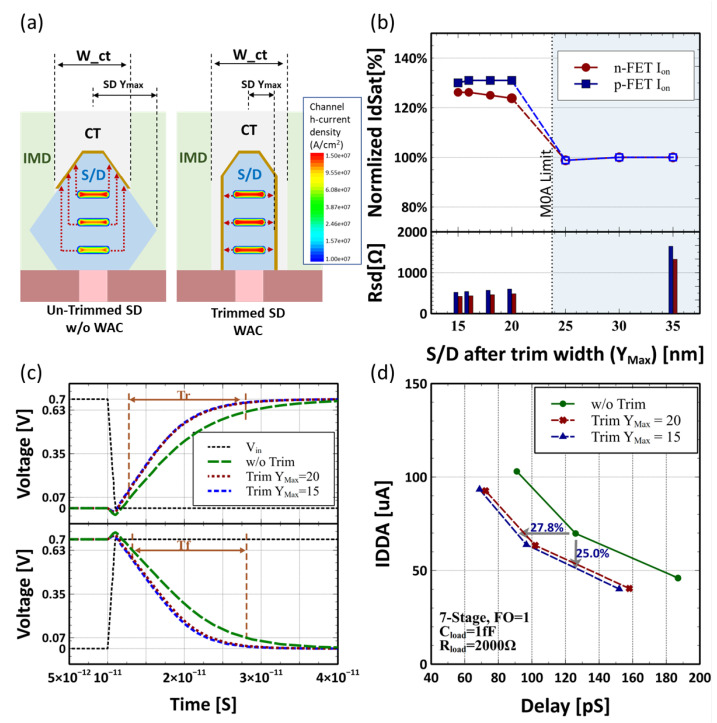
(**a**) Current from bottom NS of trimmed S/D and WAC has much a shorter distance to contact compared to an un-trimmed case, resulting in a more equally distributed current between all three nanosheets and better efficiency; (**b**) ion performance and extracted Rsd at varied S/D trimming position; (**c**) switch delay of an inverter on a fixed load of 1fF. Both Tr and Tf of NS-FETs reduce with S/D trimming; (**d**) power–delay curve of a constructed 7-stage ring oscillator.

Besides the suppression of parasitic capacitance, meanwhile, the S/D trimming process could potentially improve the S/D parasitic resistance. In advanced nodes, S/D access resistance plays an important role in device performance, which accounts for more than 30% of the total resistance [9]. For the 3 nm node, with an estimated cell height of 115.5 nm and N/P separate distance 20.5 nm [10], the maximum width of M0A contact is limited to 47.5 nm. As shown in Figure 3a, limited by the width of M0A contact ($W_{ct}$), for large diamond shaped S/D, the contact can only land on the top of S/D, which causes large access resistance, especially for the bottom nanosheet channel. With the S/D trimming, wrap-around-contact (WAC) technology would be feasible and can make all the stacked nanosheets electrically equivalent as illustrated in Figure 3a. The quantitative impact has been simulated as in Figure 3b, showing for S/D trimming to Ymax∼20 nm, the transistor driving current can be boosted by 31% and 24% for p-FET and n-FET, respectively. With trimming to Ymax∼15 nm, n-FET drive current gains an additional ∼3% due to further reduction of access resistance. Meanwhile, p-FET drive current almost remains unchanged, mainly due to the small loss of channel stress.

To examine the AC performance with S/D trimming, inverter and 7-stage ring oscillator (RO) [11] are evaluated by plugging in the device’s model generated by Primarius BSIMplus [10] . The inverter switch behavior is shown in Figure 3c, showing rising/falling time (Tr/Tf) improved by 49% and 53%, respectively. The IDDA-Delay plot of the 7-stage RO in Figure 3d demonstrates that the S/D trimming scheme could save up to 25 percent of power or 27.8 percent of delay. All these are attributed to the reduction of parasitic S/D RC and preservation of device drive current.

### 3.3. More Vertical Stacking of Nanosheets

**Figure 4:**
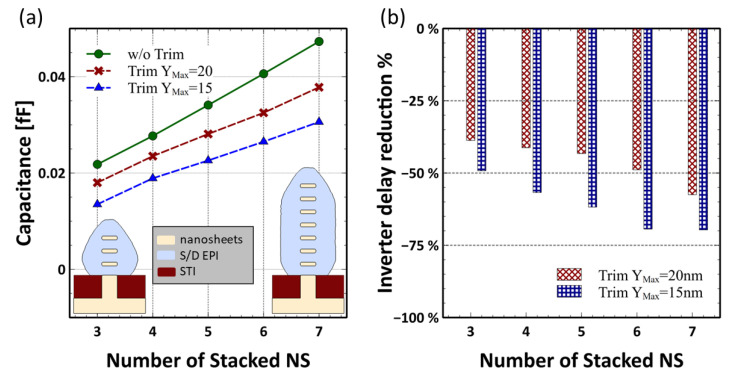
(**a**) NS-FETs vertical scaling challenge: under the same EPI growth condition, the size of S/D increases to accommodate more stacking layers, resulting in worse parasitic RC performance. (**b**) Inverter delay reduction percentage is calculated based on the corresponding un-trimmed case. Higher stacking NS benefits more by implementing the S/D trimming process.

One of the most promising advantages of GAA NS-FETs technology is the vertical scalability [2]. However, as the stacking layers increase, the epitaxy S/D also becomes bigger. This will cause even higher access resistance and bigger parasitic capacitance, degrading the device performance [12]. As shown in Figure 4a, with the proposed S/D trimming scheme, the capacitance increase with more stacking NS becomes much slower, and the S/D after trimming can be adjusted to embrace the WAC, thus further reducing the S/D parasitic Resistance. As demonstrated in Figure 4b, as the number of stacked nanosheets increases, more benefits could be gained from the S/D trimming regarding the inverter delay. This may provide a very promising solution for S/D parasitic suppression and GAA technology extension beyond the 3 nm node.

## 4. Conclusions

By applying the proposed self-aligned S/D trimming process, the parasitic S/D RC has been significantly reduced while retaining the channel stress at most. As a result, the AC performance of inverter and RO is greatly improved. Furthermore, it enables more stacking of nanosheets for higher density and performance, which could be a key factor for GAA NS-FETs technology.
